# Molecular Screening of Nasopharyngeal Carcinoma: Detection of* LMP-1*, *LMP-2* Gene Expression in Vietnamese Nasopharyngeal Swab Samples

**DOI:** 10.31557/APJCP.2019.20.9.2757

**Published:** 2019

**Authors:** Thuan Duc Lao, Tuan Anh Hoang Nguyen, Kha Dong Ngo, Hue Hong Thieu, Minh Trong Nguyen, Dung Huu Nguyen, Thuy Ai Huyen Le

**Affiliations:** 1 *Department of Pharmaceutical and Medical Biotechnology, Faculty of Biotechnology, Ho Chi Minh City Open University, Ho Chi Minh City, *; 2 *Faculty of Biology and Biotechnology, University of Science, VNU-HCM, *; 3 *Cho Ray Hospital, HCMC, Vietnam.*

**Keywords:** LMP-1, LMP-2, Epstein-Barr virus, nasopharyngeal swab sample, Vietnam

## Abstract

**Objective:**

This study aimed to characterize the expression of* LMP-1*, *LMP-2* in clinical swab samples in order to find out the potential molecular based biomarker for NPC diagnosis and screening, which could offer a chance in development of rapid method for NPC diagnosis in Vietnamese population.

**Materials and Methods:**

A total of 93 nasopharyngeal carcinoma swab samples and 100 healthy nasopharyngeal swab samples were collected to evaluate* LMP-1*, *LMP-2* expression by Real-time reversed PCR.

**Results:**

we figured out the significant association between the expression of* LMP-1* (counting for 48.39%), *LMP-2* (counting for 39.78%) and NPC. No *LMP-1* expression was observed, and only 1 of 100 specimens was detected with LMP-2 positive in healthy samples. In the combination of* LMP-1* (+) and/or* LMP-2* (+), the frequency of positive was 53.76%, greater than each gene expression. Additionally, sensitivity, specificity, positive predictive value, negative predictive value of assay were 99.00%, 98.04%, 69.72%, and 77.02%, respectively. Additionally, the *LMP-2* expression level was 5.50 times higher in NPC samples than non-cancerous samples.

**Conclusion:**

Our results indicated the molecular invasive method based on the expression of* LMP-1*, *LMP-2* in swab samples would be a promising supplement in NPC diagnosis, screening in the near future in Vietnam.

## Introduction

A well-established etiology factor of NPC is its strong associated to Epstein-Barr virus (EBV), also known as human gamma herpes virus 4 (HHV4), has been postulated since the year of 1966 (Rowe et al., 1992; Vera-Sempere et al., 1996; See et al., 2008, Young, Dawson, 2014; Mahdavifar et al., 2016). EBV remains two alternative lifestyles: (1) the latency stage; (2) the lytic stage or productive phrase of EBV (Tsurumi et al., 2005). Between two stages, EBV establishes life-long latent persistence (Bocian, Januszkiewicz-Lewandowska, 2011). In latency state, EBV genes are expressed in the limited set of viral latent transcripts (Young et al., 2000; Raab-Traub, 2002; Marquitz and Raab-Traub, 2012). Among the latent genes products,* LMP-1* (*Latent membrane protein-1*) and* LMP-2 *(*Latent membrane protein-2*) are consistently expressed in subset of NPC tumors, therefore, they display useful oncogenic targets of NPC diagnosis (Brooks et al., 1992; Hao et al., 2004; See et al., 2008; Lao et al., 2017).* LMP-1* encoded its latent membrane protein that induce morphological and phenotypic alterations in epithelial cells (Hao et al., 2004; Kang, Kieff, 2015; Dawson et al., 2012). The function of LMP-2 is considered as playing important role in carcinogenesis by driving EBV into latency (Thompson, Kurzrock, 2004; Dawson et al., 2012). Many previous studies showed that* LMP-1*,* LMP-2* has been considered as the molecular prognostic, diagnostic as well as the outcome of therapy in NPC (Hao et al., 2004; Hariwiyanto et al., 2010; Rosales-Pérez et al., 2014; Lao et al., 2017).

**Table 1:** The Sequences of Primers Used in Current Study

Primer	Sequences (5’ – 3’)
LMP-1-F	CAGTCAGGCAAGCCTATGA
LMP-1-R	CTGGTTCCGGTGGAGATGA
LMP-2-F	AGCTGTAACTGTGGTTTCCATGAC
LMP-2-R	GCCCCCTGGCGAAGAG

F, Forward primer; R, Reverse primer

**Table 2:** Characteristics of NPC Patients

Variables		n (%)
Sex	Male	68 (73.12)
	Female	25 (26.88)
Age	≤ 20	1 (1.08)
	20 to ≤ 40	17 (18.28)
	40 to ≤ 60	43 (46.23)
	60 to ≤ 80	31 (33.33)
	> 80	1 (1.08)
Pathological classification	Type 1	4 (4.30)
	Type 2	26 (27.96)
	Type 3	63 (67.74)
Stage	I	0 (0.00)
	II	33 (35.48)
	III	15 (16.13)
	IV	45 (48.39)

Type 1, keratinizing squamous cell carcinoma; Type 2, non- keratinizing carcinoma; Type 3, undifferentiated carcinoma

**Table 3:** Data Analysis of *LMP-1, LMP-2*

	*LMP-1*		*LMP-2*		RPI ≥ 0.5	
	P (%)	N (%)	P (%)	N (%)	P (%)	N (%)
NPC	45	48	37	56	50	43
(n = 93)	(48.39)	(51.61)	(39.78)	(60.22)	(53.76)	(46.24)
Control	0	100	1	99	1	99
(n = 100)	(0.00)	(100.00)	(1.00)	(99.00)	(1.00)	(99.00)
p	< 0.0001		< 0.0001		< 0.0001	

P, positive, N, negative

Vietnam, located in Southern Asia, is well known as the high incidence and mortality rate of nasopharyngeal carcinoma within 86,691 cases (Age-standardized rate – ASR = 1.2/100,000) and 50,831 (ASR = 0.7/100,000) deaths (Mahdavifar et al., 2016; Globocan, 2012). In our previous, we reported our experience of detecting EBV-derived genomic DNA, includes EBNA-1, EBNA-2,* LMP-1* and* LMP-2*, counting for 46.32%, 49.47%, 45.26% and 47.37%, respectively, by nasopharyngeal swab in NPC patients (Lao et al., 2017). Moreover, low frequencies of those genes were observed in non-cancerous samples. We also proved that the *EBNA-1*, *EBNA-2*,* LMP-1*, *LMP-2* based detection are useful oncogenic targets to screen NPC (Lao et al., 2017). EBV infection in NPC has been classified as the latency type 2 infection in which only *EBNA-1*, *LMP1*, *LMP2* expressions could be detected (Korcum et al., 2006). It may throw light on the development of mRNA EBV expression based molecular screening, diagnosis, prognosis for NPC in the high-incidence areas, includes Vietnam. Continuing with our previous study, for the aims to find out the potential molecular biomarkers for NPC management, we defined the EBV latency-derived* LMP-1*,* LMP-2* gene expression whether or not associated with NPC in Vietnamese NPC patients. Especially, we carried out on nasopharyngeal swab samples, the non-invasive samples, and it would be a good supplement and promising role in the further NPC screening and diagnosis. 

## Materials and Methods

*Ethics statement, samples collection*

Institutional Ethics Board approval was obtained from the Medical Ethics Committee of the Cho Ray Hospital, Ho Chi Minh City, Vietnam. (The decision number of the permission from Ethical committee: 516/BVCR-HDDD, Cho Ray Hospital, Ho Chi Minh City, Vietnam). All the samples used in this study were agreed by Cho Ray Hospital and obtained from all participants in current study. The patient, who was enrolled in this study, are required to be agreed and sign on the consent forms to approve the usage of the samples for laboratory work and analysis.

A total of 93 NPC swab samples, confirmed by immunohistochemistry, were archived and admitted from the Cho Ray Hospital, Vietnam. For 100 non-malignant swab samples, which were negative for nasopharyngeal carcinoma, were collected from non-NPC patients. In brief, a 15-cm-long cotton stick was inserted into the nasal cavity and moved toward the nasopharyngeal wall, then, swept over the surface of the posterior and lateral nasopharyngeal wall. The cotton stick was withdraw and immediately immersed in the phosphate-buffered saline solution, stored at -20^o^C for further experiments.

Total RNA extraction, real-time PCR assay

Total of RNA was extracted by using TRIzolTM Reagent (Cat: 15596026, Invitrogen). cDNA was reverse transcribed from approximately 5 ng of Total RNA by using The High Capacity cDNA Reverse Transcription Kit (Thermo Fisher Scientific). All the RNA extraction and the reverse transcriptions assays were performed according to the manufacturer’s instructions. For cDNA* LMP-1*,* LMP-2* detection, qRT-PCR reactions were done by means of a qSYBR-green, and GAPDH was used as an endogenous control. The internal control candidate was used to normalize the Ct values of each* LMP-1*,* LMP-2*. The primers, which were used in current study, were obtained by previous study (Table 1).

*Statistical analysis*

Data were analyzed using Medcalc^®^ Version 12.7.0.0. All p values were two-side, and less than 0.05 were considered statistically significant. All values were reported as mean ± SEM. The relative expression of* LMP-1*, *LMP-2* as determined using q-PCR was analyzed using the 2^-ΔΔCt^ method. Finding was greater and less than 1 was determined to classify up-regulation and down-regulation, respectively. Chi-test was used to determine the association between the expression of* LMP-1*,* LMP-2* and NPC status. Moreover, the association between expression of *LMP-*1, *LMP-2* and risk of NPC was estimated by computing OR, RR and 95% confidence intervals (CI).

## Results

*Patient characteristics*

The characteristics of the total of 93 NPC patients were summarized in Table 2. The mean age of 93 NPC case was 53.51 ± 1.43 (range: from 20 to 81). Among them, the number of male (counting for 73.12%) is more than female (counting for 26.88%) by 2.72 times. The age incidence profile indicated the increase of NPC risk by the age up to the late middle age (range: from 40 to under 60). Tumor histological types were classified according to World Health Organization (WHO) classification for NPC criteria. Type 3 (undifferentiated carcinoma: UC) counted for the highest proportion of all types of NPC. 67.74% (n = 63) NPC cases were classified as type 3 (undifferentiated carcinoma), occupied the highest proportion of its cases. Stage wise, 48.39% (n = 45) NPC patients was observed in an advanced stage, and no case was observed in the early stage (stage 1).

*Epstein-Barr virus genes: LMP-1, LMP-2 expression were highly expressed in NPC clinical swab samples*

The frequencies of* LMP-1*, *LMP-2* expression were shown in Table 3. In the NPC swab set, 45 of 93 NPC specimens (counting for 48.39%) were positive for *LMP-1* expression, whereas none of the controls was positive. 37 of 93 NPC samples (counting for 39.78%), 1 of 100 non-cancerous samples (counting for 1.00%) were positive for LMP-2 expression, respectively. Table 3 showed the validity of expression each gene in NPC clinical swab samples. A value of p < 0.0001 indicated that the each gene expression were significantly associated with NPC. Using the* LMP-1* gene and later rectification with* LMP-2* expression [*LMP-1* (+) and/or *LMP-2* (+): RPI (Real-time PCR Index) ≥ 0.5, meant that at least one of two genes were expressed], NPC could be diagnosed with a sensitivity of 53.76% (50/93), specificity of 99.00% (99/100), positive predictive value of 98.04% (50/51), negative predictive value of 69.72% (99/142), and accuracy of 77.02% (149/193). No significant association was found between* LMP-1*,* LMP-2* expression and other clinical characteristics, such as patient’s gender, age, tumor histological types, as well as stage (p > 0.05).

No significant association was found between* LMP-1*, *LMP-2* expression and other clinical characteristics, such as patient’s gender (LMP-1: p = 0.06; LMP-2: p = 0.83), age (*LMP-1*: p = 0.19; *LMP-2*: p = 0.26), tumor histological types (*LMP-1*: p = 0.29; *LMP-2*: p = 0.28), as well as stage (*LMP-1*: p = 0.07; *LMP-2*: p = 0.40).

The mean Ct values for GAPDH in the case and control group were 27.14 ± 2.31 and 27.75 ± 2.24, respectively. No significant difference in the GAPDH expression was found between case and control group (p = 0.77 > 0.05). Therefore, GAPDH was suitable as a reference gene (internal control) to normalize the* LMP-1* and *LMP-2* expression between the case and control groups. The meant Ct value of* LMP-1* in the case was 25.14, and no Ct value was recorded due to no control samples were positive for *LMP-1* expression. Although LMP-1 was not detected in control group, it also could be confirmed that the expression of* LMP-1* was elevated in patient samples. The relative expression of *LMP-2 *was computed by the 2^-ΔΔCt^ method based on the comparison of *LMP-2* expression in case and control group. The mean of Ct value of *LMP-2 *in the case and control group were 24.93 and 27.97, respectively. As the result, the *LMP-2* expression level was 5.50 times higher in NPC samples than non-cancerous samples (2^-ΔΔCt ^= 5.50, p < 0.05). 

## Discussion

Up to date, there are challenging to diagnosis NPC, because of the vague symptoms such as hearing loss, bloody nasal discharge, diplopia and headache. Especially, in Vietnam, the high incidence and mortality rate of NPC, most patients present at an advance stage when first diagnosis (Stage 3, or stage 4). There is essential to find an early diagnosis and biomarker to achieve favorable treatment and increasing of patient’s survival. Many previous studies have been reported that the association between NPC and EBV may throw light on the molecular diagnosis of NPC (Hildesheim and Levine, 1993; Hao et al., 2004; Lao et al., 2017; Wu et al., 2018). According to the function of* LMP-1*, its encoded protein plays a key role as a viral mimic of the TNFR family member, CD40, engaging a number of signaling pathways, such as NF-κB, JNK, p38 pathway (Hao et al., 2004; Xu et al., 2006; Dawson et al., 2012; Kang and Kieff, 2015). LMP-2 plays important role in carcinogenesis by driving EBV into latency and provides essential survival signals through the constitutive activation of the ERK/MAPK pathway (Thompson and Kurzrock, 2004; Dawson et al., 2012). In the latent stage, *LMP-1*,* LMP-2* expression, which are expressed in EBV latency type II, exemplified by NPC, contribute to cell survival (Kang and Kieff, 2015). Thus,* LMP-1* and *LMP-2* are thought to be the meaningful biomarker for NPC management.

The use of NPC biopsy samples were considered as the “standard” sample in NPC diagnosis, based on imaging diagnosis, immunohistochemistry, etc. However, the non-invasive samples, such as peripheral blood, nasopharyngeal swabs, throat swabs, plasma and saliva have been used for detecting the etiological causes of NPC, including the presence of EBV (Liu et al., 2013; Zheng et al., 2015; Lao et al., 2017). Therefore, in current study, the nasopharyngeal swab samples were used in the detection of *EBV LMP-1*, *LMP-2* expression to find out the non-invasive, potential biomarker for NPC diagnosis and screening. 

In current study, this is the first case – control study was carried out to evaluate whether or not* LMP-1*, *LMP-2* expression could be further applied in NPC screening, diagnosis as well as therapy in Vietnam. As the result, we pointed out the significant association between the expression of* LMP-1* (counting for 48.39%), LMP-2 (counting for 39.78%) and NPC. Whereas, in the case of control group, no *LMP-1* expression was observed, and only 1 of 100 specimens was detected with *LMP-2* positive (p < 0.0001). Additionally, the LMP-2 expression was confirmed to be 9.78 times higher in tumor samples, compared to non-cancerous samples (2^-ΔΔCt^ = 5.50, p < 0.05). Although* LMP-1* was not detected in control group, it also could be confirmed that the expression of* LMP-1* was elevated in patient samples. In the combination of *LMP-1* (+) and/or* LMP-2* (+) (RPI (Real-time PCR Index) ≥ 0.5), the frequency of positive was 53.76%, greater than each gene expression. Additionally, sensitivity, specificity, positive predictive value, negative predictive value of assay were 99.00%, 98.04%, 69.72%, and 77.02%, respectively. Thus, it indicated that the oncogenic role of* LMP-1*, *LMP-2* in NPC was well evaluated and confirmed in this case – control group study. Therefore, our investigations reveal that *LMP-1*, *LMP-2* expression-based invasive method was identified as further promising biomarker for prognosis, diagnosis and therapy for NPC. 

In conclusion, in current study, we found out the significant association between* LMP-1*, *LMP-2* expression as well as* LMP-1* and/or* LMP-2* expression and NPC in Vietnamese NPC patients. Our results pointed out the significant association between* LMP-1*, and* LMP-2* expression and NPC, performed by Real-time reverse PCR method. This molecular-based invasive method would be a promising supplement in NPC diagnosis, screening in the near future in Vietnam.
